# Supplementary material for: Role of miRNAs in Human T Cell Leukemia Virus Type 1 Induced T Cell Leukemia: A Literature Review and Bioinformatics Approach
Source: Int J Mol Sci. 2022 May 14;23(10):5486. doi: 10.3390/ijms23105486 (PMC9141946; doi:10.3390/ijms23105486)
Supplement: Supplementary file 1 [file ijms-23-05486-s001.zip › Supplementary Figure S1.pdf]

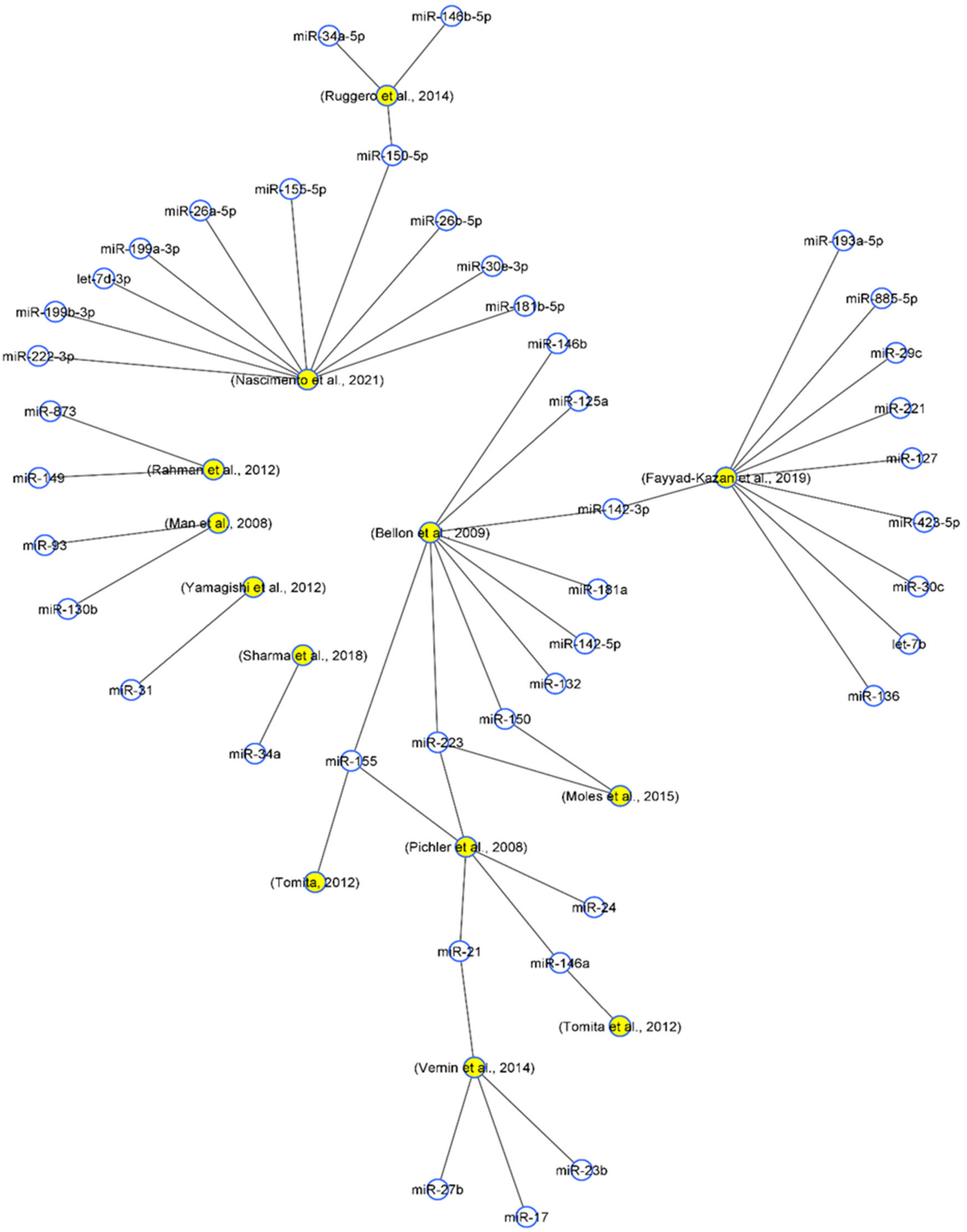

Supplementary Figure S1. Interaction network between miRNAs and their respective references retrieved in PubMed built with Cytoscape. The yellow nodes represent the studies found in the literature connected with the respective associated miRNAs. The network topology was constructed with 55 nodes and 51 edges with a density metric equal to 0.017.
